# The cost-utility of maintenance treatment with venlafaxine in patients with recurrent major depressive disorder

**DOI:** 10.1111/j.1742-1241.2008.01711.x

**Published:** 2008-04-01

**Authors:** P Sobocki, M Ekman, A Ovanfors, R Khandker, B Jönsson

**Affiliations:** 1 Department of Learning, Informatics, Management and Ethics, Medical Management Centre, Karolinska Institutet Stockholm, Sweden; 2 European Health Economics Stockholm, Sweden; 3 i3 Innovus Stockholm, Sweden; 4 Wyeth AB Stockholm, Sweden; 5 Wyeth Research Collegeville, PA, USA; 6 Stockholm School of Economics Stockholm, Sweden

## Abstract

**Aims:**

The Prevention of Recurrent Episodes of Depression with venlafaxine XR for Two Years trial has reported advantages with maintenance treatment for patients with recurrent depressive disorder. The aim of this study was to assess the cost-utility of maintenance treatment with venlafaxine in patients with recurrent major depressive disorder, based on a recent clinical trial.

**Methods:**

A Markov simulation model was constructed to assess the cost-utility of maintenance treatment for 2 years in recurrently depressed patients in Sweden. Risk of relapse and recurrence was based on a recent randomised clinical trial assessing the efficacy and tolerability of maintenance treatment with venlafaxine over 2 years. Costs and quality of life estimations were retrieved from a naturalistic longitudinal observational study conducted in Sweden. Health effects were quantified as quality-adjusted life-years (QALYs). Sensitivity analyses were conducted on key parameters employed in the model.

**Results:**

In the base-case analysis, the cost per QALY gained of venlafaxine compared with no treatment was estimated at $18,500 over 2 years. In a probabilistic sensitivity analysis, we found that maintenance treatment with venlafaxine is cost-effective with 90% probability at a willingness to pay per QALY of $67,000 or less. Our long-term analyses also indicate that even under conservative assumptions about future risks of recurrences, maintenance treatment is cost-effective.

**Conclusion:**

The present study indicates that maintenance treatment for 2 years with venlafaxine is cost-effective in patients with recurrent major depressive disorder.

**What's known**

- Depression may develop into a recurrent disease, and the risk of recurrences seems to increase with the number of previous episodes of depression.
- Full remission from depression is associated with significantly lower costs and higher quality of life than no or only partial response.
- Hence, an important treatment goal is to achieve remission and prevent recurrences.

**What's new**

- This is the first cost-utility study of long-term maintenance treatment with venlafaxine in patients with recurrent unipolar major depression.
- The results indicate that maintenance treatment for 2 years in recurrent depression is cost-effective.

## Introduction

Depression is one of the most important public health problems in the industrialised world, and is associated with a substantial economic burden on society. In the USA, Greenberg et al. (1) found a total cost of 83.1 billion USD for the year 2000, whereof 31% direct medical costs, 7% suicide-related mortality costs and 62% costs for lost productivity at work. The overall prevalence was estimated at 18.1 million cases, and the treated prevalence was estimated at 7.9 million patients, for the year 2000.

There is a growing awareness of the need for a long-term perspective in the treatment of affective disorders (2–6). In many patients depression may develop into a recurrent disease, and the risk of recurrences seems to increase with the number of previous episodes of depression (7). It is therefore an important treatment goal to prevent recurrences.

Full remission means improvement to the degree that the patient is asymptomatic, i.e. has no more than minimal symptoms. In this context it is fruitful to make a distinction between relapse and recurrence (8). A relapse occurs if the depressive symptoms return relatively quickly after an initial remission from a depressive episode. If the patient has stayed in full remission for a period long enough to qualify as recovery, and depressive symptoms then come back, it is a recurrence. Accordingly, treatment can be divided into three stages: acute treatment phase, continuation therapy to prevent relapse and maintenance therapy to prevent recurrence (9).

A key question is for how long the patient should remain on maintenance treatment after achieving remission from an acute episode of depression. It is often recommended that patients should be treated for 4–6 months after going into remission (4,9), but there is no wealth of studies with such a long or longer follow-up after the acute treatment phase. An extensive meta-analysis has been published which establishes the benefits of antidepressant drug treatment on the relapse risk (9). Most of the individual studies used as a basis for the analysis are too short for covering also the long-term recurrence risk, but the results of the meta-analysis indicate that there is no clear distinction between the continuation and maintenance treatment effects.

Also from a health economic viewpoint it is important to take full remission and the risk of recurrence into account. Not surprisingly, full remission is associated with significantly lower costs and higher quality of life than no or only partial response (10). The highest costs are for sickness absence (about two-thirds), while the costs for antidepressant treatments are only 6–8% of the total costs (11).

The purpose of the present study was to investigate the cost-utility of 2-year maintenance treatment with venlafaxine in patients with recurrent unipolar major depression in the Swedish healthcare setting. The analysis was based on a clinical trial comparing venlafaxine to placebo (12).

## Methods and materials

### Cost-effectiveness analysis

The incremental cost-effectiveness ratio (ICER) is defined as:
(1)
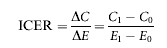

where Δ*C* is the difference in total cost between intervention and no intervention, and Δ*E* is the difference in effectiveness between intervention and no intervention.

Costs can be divided into two different categories: direct and indirect costs. Direct costs included in the present study include costs because of hospitalisations, outpatient visits and drugs. Indirect costs are costs related to lost productivity because of the illness. In this study, quality-adjusted life-years (QALYs) will be used as an outcome measure, as it is the most relevant measure of effectiveness from a health-policy perspective.

### The model

**Figure 1:**
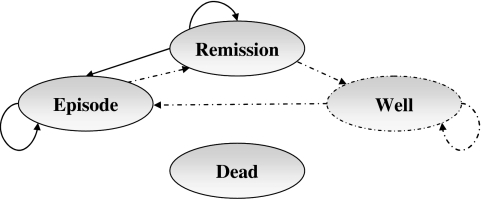
Structure of the Markov cohort simulation model

Cost-effectiveness analysis in depression generally requires modelling, as all the required data are seldom available from a single dataset over the relevant timeframe. For the present analysis, a Markov model was developed in line with the design of the Prevention of Recurrent Episodes of Depression with VENlafaxine XR for Two Years study (4), and is a modified version of a previously published model (13). The structure of the model is shown in the state transition diagram in Figure 1. In a Markov model, the patients are classified into a number of different health states, each associated with a certain cost and utility. As time progresses in the model, the patients can move between different states (depressive episode, remission, well and dead) according to a set of transition probabilities. Patients may move from one health state to another during a defined interval of time called a cycle.

The cycle length was set to 1 month and all patients are followed through the model for 2 years (equal to the length of the clinical trial). Given the disease characteristics and the 2-year follow-up time, monthly intervals were appropriate for the cycle length in the Markov model, because this is a clinically meaningful cycle length and gives good precision in the model. There is always a probability to remain in the same state or to die. All the patients begin in the remission health state, because that is the starting point of the maintenance treatment phase. Each month a patient has a probability of relapsing or to die. If a patient dies, he will move to the dead health state and remain there for the rest of the simulation (arrows to the dead health state were excluded to simplify the figure). If the patient relapses he will move to the relapse state. In the base case, patients were not allowed to remit from a relapse during the maintenance treatment phase, to model the cost-effectiveness of venlafaxine as close to the clinical trial design as possible. However, in sensitivity analysis patients were allowed to remit from a relapse, turn well after 6 months of being in the state remission, and recur to a depressive episode after having recovered, i.e. from being in state well (see dotted arrows in Figure 1).

### Clinical trial data

The present analysis was based on a recent clinical trial (12). The trial aimed at assessing the efficacy and tolerability of venlafaxine as maintenance treatment in recurrent depressive patients. The double-blind randomised multicentre trial started with a 10-week treatment period during the acute phase of depression, followed by a 6-month continuation phase for patients who responded to treatment [17-item Hamilton Rating Scale for Depression (HAM-D_17_) total score ≤ 12 or 50% decline from baseline] and remitters (HAM-D_17_ ≤ 7). Patients treated with venlafaxine, and who had satisfactory response or remission also after the continuation phase, were randomised 1 : 1 between venlafaxine and placebo in a maintenance phase. The maintenance phase was in turn divided into two consecutive 12-month periods, A and B. At the end of maintenance period A, patients who continued to respond to venlafaxine were once again randomised 1 : 1 between venlafaxine and placebo in maintenance period B. The results from the trial were based on intention to treat.

The purpose of the maintenance phase was to investigate the effect of maintenance treatment with venlafaxine on the recurrence rate compared with placebo. Placebo is a relevant comparator in this phase, as the optimal duration of maintenance treatment is still an issue for debate.

The inclusion criteria in the study were age ≥ 18 years, fulfilling the Diagnostic and Statistical Manual of Mental Disorders criteria for major depressive disorder with at least two episodes of major depression, excluding the current one, in the past 5 years and with at least 3 months between the end of the previous episode and the beginning of the present one. The patients should also have had depressive symptoms for at least a month prior to randomisation, with a HAM-D_17_ score ≥ 20 at screening, and a HAM-D_17_ score larger than 18 at randomisation.

Patients who failed to respond to venlafaxine during the acute (and later phases) discontinued the study. Patients with a history of treatment resistance or psychiatric comorbidities such as bipolar disorder and eating disorder were excluded, as were patients with a primary diagnosis of panic disorder, obsessive compulsive disorder, generalised anxiety disorder, social phobia or posttraumatic stress disorder within 6 months prior to screening.

The primary efficacy measure was the HAM-D_17_ scale, which was administered at each monthly visit. The primary endpoint of the study was time to recurrence of depression, with recurrence defined as a HAM-D_17_ score > 12 and a HAM-D_17_ score reduction of at most 50% compared to acute-phase baseline at two consecutive visits.

Secondary efficacy measures included rate of response (HAM-D_17_ total score ≤ 12 or 50% decline from baseline), remission (HAM-D_17_ ≤ 7), Clinical Global Impression – Severity scale (CGI-S), Medical Outcome Short Form (36) Health Survey, and some other standard scales.

### Target patient group

The target patient group included in the model was based on the study population included in the clinical trial (12). These patients were high-risk patients with recurrent depressive episodes. Patients included in the analysis had been successfully treated with antidepressant therapy for a depressive episode, and had remitted from the episode with a HAM-D_17_ score of ≤ 7, which is a generally accepted level of clinical remission in depression (14). The mean age of the study sample was 42 and 67% of the sample were women. On average, the patients were moderately depressed at inclusion to the clinical trial (CGI-S mean score of 4.3 or HAM-D_17_ score of 22).

### Health economic data

Cost data for the ‘episode’ and ‘remission’ health states in the model were retrieved from the naturalistic observational study ‘Health Economic Aspects of Depression In Sweden’ (HEADIS) conducted in Swedish primary care (11). The patient characteristics of the HEADIS study corresponded well with the study population of the clinical trial, with a mean age of 47, 67% women, and a CGI-S mean score of 3.9 (11), which means that the costs from this study should be representative also for patients with the characteristics in the clinical trial. Cost data were incorporated both from a healthcare payer and a societal perspective. In the healthcare payer perspective, the costs correspond to the amount paid or reimbursed within the healthcare system. The societal cost includes the total expenditure because of the disease, regardless of who covers the cost. This includes costs both for health care and for sick leave paid by employers and social insurance funds. Data on resource use included primary care visits, hospital visits and visits to other health professionals (e.g. psychologists and counsellors). Productivity costs included productivity lost because of absenteeism. Data on quality of life for depressive episode and remission, measured with the EuroQoL-5D health status questionnaire, were retrieved from the HEADIS study (15), and was used to estimate QALYs in the model. The data from the HEADIS study showed that remission was an important predictor of health-related quality of life, whereas other demographic and clinical variables were not statistically significant (10,15). The results from the HEADIS study are thus applicable to use for the health states ‘Episode’ and ‘Remission’ in the Markov model (10). Adjustments for multiple comparisons were not performed for the parameter estimations, as point estimates were used in the cost effectiveness model. In the sensitivity analysis, the model was extended, which allowed for patients ending up in the health state ‘Well’, and a mean utility score was taken from a study of the health-related quality of life in the general population conducted by Burström et al. (16). The cost and utility values applied in the model are summarised in Table 1.

**Table 1:** Data included in the model

Parameter	Data (95% CI)	Source
**Costs by states**	($/month)	
Well*	0	
**Episode**		
Direct healthcare costs	433 (382–518)	(10)
Indirect costs	938 (774–1108)	(10)
**Remission**		
Direct healthcare costs	273 (157–341)	(10)
Indirect costs	555 (437–681)	(10)
Dead	0	
**Health utility weights**		
Well*	0.86 (SE 0.009)	(16)
Episode	0.57 (0.52–0.61)	(10)
Remission	0.81 (0.78–0.84)	(10)
Dead	0	
**Transition probabilities**		
Risk of relapse	Survival function	(4)
Probability of remitting*	Survival function	(10)
Risk of re-relapsing*	0.15	(9)
Risk of recurrence (episodes/year)*	0.20	(19)
Increased risk of recurrence with previous episodes (hazard ratio)	1.15 (1.11–1.18)	(20)
Increased risk of death with depressive episode (SMR)	20.4 (SE 1.1)	(18)

* Included in the sensitivity analysis. CI, confidence interval; SMR, standardised mortality ratio.

### Cost of intervention

The cost of venlafaxine was based on mean dosages given in the clinical trial and drug prices listed in the National Pharmaceutical Drug Price list (http://www.fass.se). Venlafaxine was administered at an average dosage of 217 mg the first year and 200 mg the second year. A weighted daily drug cost was calculated for both periods of the 2-year maintenance treatment phase.

### Cost of adverse effects

The clinical trial reported no significant differences in adverse effects between the treatment arms. However, costs because of the most common adverse effects were included in the model in the sensitivity analysis.

### Transition probabilities

#### Risk of relapse

The relapse risk of a new episode during the maintenance treatment period was based on the combined follow-up data retrieved from the clinical trial (12). Relapse was defined as a HAM-D_17_ score of ≥ 12. A Weibull regression model was estimated on survival data from the clinical trial (Wyeth, data-on-file) measuring time to relapse between the two treatment arms (Table 2). The Weibull distribution was chosen as it is suitable for modelling data with hazard rates that increase or decrease over time, and allows for the estimation of the probability of an event in different time intervals after the starting point. The monthly risk of relapse was, thereafter, estimated and employed in the Markov model.

**Table 2:** Weibull survival function on time to relapse comparing venlafaxine with placebo (months), no hazard

	Coefficient	SE	z	P > *z*	95% CI	
Venlafaxine*	−0.546	0.281	−1.94	0.052	−1.097	0.004
Constant	−2.561	0.263	−9.74	0	−3.076	−2.045

* A dichotomous variable was included: venlafaxine for 1 and placebo for 0. LR χ_1_^2^=3.84; p = 0.05. LR, likelihood ratio.

#### Probability of remitting

In the base case it was not taken into account that patients could remit from a relapse in the maintenance treatment phase. As a sensitivity analysis, the model was extended to allow for this, and monthly estimates of probabilities of remitting were derived from the naturalistic observational study HEADIS (10).

### Mortality risk

Mortality rates were taken from the general population in Sweden (17), and based on the literature it was assumed that patients having a depressive episode had an increased relative risk of dying because of suicide of 20.4 (18).

### Other data

In a sensitivity analysis it was also allowed for re-relapses, recovery (health state well) and recurrence. Geddes et al. (9) have conducted a thorough meta-analysis based on clinical trials, and estimated the risk of relapse to be 0.15 during 6 months of treatment with antidepressants. The risk of recurrence was set to 0.20 per year (19). However, the risk was assumed to increase with the number of previous episodes (hazard ratio 1.15) (20).

### Analysis

#### Base-case analysis

Costs were reported both from the societal perspective and from the healthcare perspective. The difference is that the former also includes productivity losses because of absenteeism from work. All costs were reported in year 2005 values and given in US dollar currency ($1 = SEK7.5). As recommended by most national pharmaceutical benefits boards a yearly discount rate of 3% was used for both costs and effects (21). Effects were measured in terms of QALYs. In the base case, the time frame of the analysis was set equal to the maintenance treatment phase of the clinical trial.

#### Stochastic analysis

To capture some of the uncertainty in the underlying parameters a stochastic analysis was performed for the base-case scenario. Parameters included in the stochastic analysis are listed in Table 3. Where patient-level data was available (costs, quality of life and risk of relapse) the statistical bootstrapping method was employed (1000 replications) using the bias-corrected accelerated bootstrap method (22), as suggested by previous researchers (23). The stochastic analyses were based on Monte-Carlo simulations with 10,000 replications.

**Table 3:** Parameters given measures of uncertainty in the probabilistic analysis

Parameter	Source	Method
**Costs by states**		
**Episode**		
Direct healthcare costs	(10)	Bootstrapping mean estimate
Indirect costs	(10)	Bootstrapping mean estimate
**Remission**		
Direct healthcare costs	(10)	Bootstrapping mean estimate
Indirect costs	(10)	Bootstrapping mean estimate
**Quality of life**		
Episode	(10)	Bootstrapping mean estimate
Remission	(10)	Bootstrapping mean estimate
**Transition probabilities**		
Risk of relapse	(4)	Bootstrapping Weibull regression
**Mortality**		
Risk of death (SMR)	(18), (17)	Normal distribution

SMR, standardised mortality ratio.

### Sensitivity analysis

The following key parameters were tested for in the sensitivity analysis: the model time frame (varied from 6 months to 4 years), discount rates (varied from 0% to 10%), drug acquisition cost (varied by ±50%), cost of adverse effects, relapse risk (varied ±20%), and mortality risk (varied ±20%). Furthermore, the Markov model developed for the within-trial analysis was extended to allow for re-recurrences and recovery and to be able to project the cost-effectiveness of maintenance treatment over long term.

## Results

### Base case

In base case, maintenance treatment for 2 years with venlafaxine was assessed compared with no treatment. From the societal perspective treatment with venlafaxine came at incremental cost of $1020 over 2 years, but generated a gain in QALYs of 0.055. This resulted in an ICER of $18,500. The corresponding results from the healthcare perspective were an incremental cost of $2000 over 2 years, a gain in QALYs of 0.055, and a cost-effectiveness ratio of $36,000 per QALY gained (Table 4).

**Table 4:** Base-case results

	ΔCosts ($)	ΔQALYs	ICER
**Venlafaxine vs. placebo**			
Healthcare perspective	1978	0.055	35,968
Societal perspective*	1020	0.055	18,548

* Societal perspective includes indirect costs for productivity losses in addition to direct healthcare costs. ICER, incremental cost-effectiveness ratio; QALY, quality-adjusted life-year.

### Stochastic analysis

**Figure 2:**
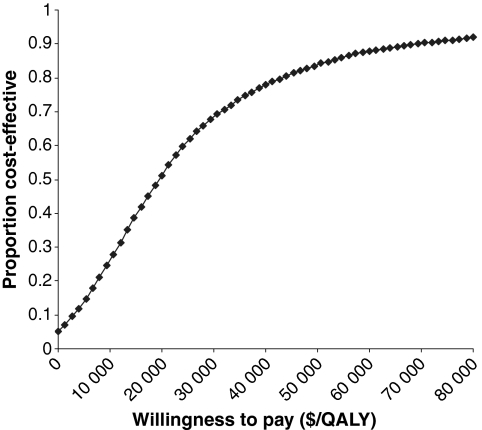
Cost-effectiveness acceptability curve. QALY, quality-adjusted life-year

In the probabilistic sensitivity analysis we assessed the uncertainty around our base-case results. The combined uncertainty in the analysis is reported as a probabilistic cost-effectiveness acceptability curve (Figure 2). At a willingness to pay for an additional QALY of $40,000, venlafaxine is cost-effective in the maintenance treatment for recurrent depression at a probability of 80%, while at a willingness to pay for an additional QALY of $67,000, it is cost-effective at a probability of 90%.

### Sensitivity analysis

In the sensitivity analysis, key parameters in the model were varied, to capture the uncertainty in the ICERs. All results from the sensitivity analysis are summarised in Table 5. In the base case, we used the data from the clinical trial for the estimation of risk of relapse during the maintenance treatment phase. Varying the relapse risk by ±20% resulted in a range of $11,900–27,600 for the cost per QALY gained. In the base case, we assumed an increased mortality rate of 20 times that of the general population. By varying this assumption by ±20%, the ICER once again ranged from $12,000 to $27,600.

**Table 5:** Sensitivity analysis

	ΔCosts ($)	ΔQALYs	ICER
**Relapse risk**			
+20%	1323	0.048	27,567
+10%	1167	0.052	22,446
Base case	1020	0.055	18,548
−10%	882	0.060	14,693
−20%	751	0.063	11,922
**Mortality risk**			
+20%	1311	0.047	27,889
+10%	1160	0.051	22,753
Base case	1020	0.055	18,548
−10%	889	0.059	15,076
−20%	768	0.064	12,000
**Simulation time frame**			
6 months	517	0.006	86,111
1 year	798	0.02	39,907
1.5 years	951	0.055	25,697
2 years*	1020	0.099	18,548
**Discount rate (costs and effects)**			
0%	1059	0.060	17,644
3%*	1020	0.055	18,548
5%	1008	0.054	18,664
10%	978	0.051	19,179
**Acquisition cost of venlafaxine**			
−50%	−169	0.055	Dominance
−30%	545	0.055	9910
−10%	782	0.055	14,225
+10%	1258	0.055	22,873
+30%	1733	0.055	31,518
+50%	2209	0.055	40,165
Costs of adverse effects†	972	0.055	17,680
Including productivitycosts at work‡	−4413	0.055	Dominance
**Extended model§**			
6 months	486	0.007	69,467
1 year	732	0.020	36,607
2 years*	1524	0.047	32,428
3 years¶	2032	0.069	29,445
4 years¶	2850	0.091	31,314

* Base-case assumptions.

† Costs were estimated for the most common adverse effects in the clinical trial, resulting in an average increased cost of $48 for the placebo group compared with patients treated with venlafaxine.

‡ Productivity cost because of reduced working capacity estimated at $4200 per month in 50% of patients in a depressive episode in the model (24).

§ Based on an extended model allowing for re-relapse and recovery and recurrence within the time frame studied.

¶ Based on maintenance treatment for 2 years and halved probability of remission for placebo patients when compared with active treatment. ICER, incremental cost-effectiveness ratio; QALY, quality-adjusted life-year.

In the base case, we employed the same time frame for the analysis as the follow-up length chosen in the clinical trial. By decreasing the treatment length and time frame for the analysis, we obtained an ICER ranging from $86,100 per QALY gained when only treating for 6 months after remission to $18,500 for 2 years (base case).

Costs and effects were discounted with an annual 3% rate in the base case. Varying the discount rates from 0% to 10%, resulted in an ICER ranging from $17,600 to $19,200 per QALY gained. When including costs associated with adverse effects from treatment, the ICER improved to $17,800 when compared with $18,500 per QALY gained in the base case. In the base case, only productivity costs because of sick leave were included in the analysis. As a sensitivity analysis, we also included productivity costs because of reduced working capacity, based on results from an American study be Stewart et al. (24), which indicated that 81% of the lost work productivity because of depression are explained by reduced performance while at work. Our results showed that venlafaxine is cost-saving compared with no maintenance treatment when reduced work performance is included.

### Long-term analysis based on extended model

**Figure 3:**
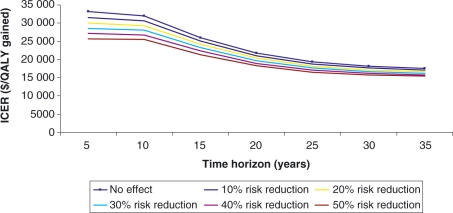
Long-term projections of the cost-effectiveness of 2-year prophylactic treatment, with varying risk reductions of future recurrences. ICER, incremental cost-effectiveness ratio; QALY, quality-adjusted life-year

In our base-case analysis, we aligned the design of the model closely to that of the clinical trial. This did, however, not allow for multiple recurrences and recovery and thus an extended Markov model was developed. The extended model resulted in ICERs varying from $69,500 with a 6-month time frame to $32,400 with a two-treatment period and time frame for the analysis. Based on the extended model long-term projections were conducted of the cost-effectiveness of prophylactic treatment. Extending the time frame of the analysis to 3 and 4 years, resulted in further decrease of the ICER (Table 5). Longer-time horizons of the analysis resulted in cost-effectiveness ratios of around $17,600 (Figure 3). Assuming that prophylactic treatment reduces the long-term risk of recurrences improves the ICER even further.

## Discussion

Based on a clinical trial assessing the efficacy of maintenance treatment with venlafaxine in recurrent depression internationally, we assessed the cost-effectiveness of this intervention. Our results indicate that, compared to not providing maintenance treatment to this high-risk group of depressed patients, venlafaxine is likely to be cost-effective within the conventional margins of willingness to pay for additional health benefits in Sweden. Our analysis indicates that the length of maintenance treatment and analysis perspective is of importance when assessing the economic benefits of maintenance treatment in this patient group.

There is no definite threshold limit regarding the highest acceptable cost per QALY gained (25). The WHO argue for international values of three times the gross domestic product per capita for developed countries (26). In the USA, threshold values of $50,000–100,000 per year of life gained have been recommended in some studies (27,28). In a survey among health economists about what threshold value to use in a cost-effectiveness analysis, Newhouse (26) reported a mean value of $60,000 per year of life gained. In Sweden, values around $90,000 (SEK650,000) have been mentioned based on the willingness to pay for saving lives on the Swedish roads (29).

The current analysis of the cost-effectiveness of maintenance treatment was based on patients having reached clinical remission from an acute depressive episode. It is widely recognised that full remission is of high importance when treating an acute phase of a depression, both from a clinical point of view (30), as well as from an economical point of view (10,13,31). However, there is a growing concern regarding the need for preventive treatment in recurrent depressive patients. Most international treatment guidelines for depression emphasise the importance of prophylactic treatment of patients with recurrent depression (32–35), suggesting that the treatment period should be at least 1–2 years. In Sweden, there are no previous national treatment guidelines in depression, but the Swedish National Board of Health and Welfare is currently developing such guidelines. However, in a recently finalised review by the Swedish Council on Technology Assessment in Health Care, it is firmly concluded that maintenance treatment is effective in recurrent depressive patients (36). The present analysis justifies not only that there are clinical benefits with maintenance treatment in recurrent depression, but also that it is likely to be cost-effective within the current levels of willingness to pay for an additional QALYs (37).

The present analysis is to our knowledge the first economic evaluation assessing the cost-effectiveness of maintenance treatment of recurrent depression in Sweden. Moreover, only a few earlier studies of this type have been conducted internationally, and no study evaluating the maintenance treatment with venlafaxine in recurrent unipolar depressive disorder. Dardennes et al. (38) conducted a cost-utility analysis of maintenance treatment in recurrent depression in France from the perspective of the French sickness fund, comparing milnacipran with no treatment over a time period of 12 months. The authors presented a cost per QALY gained at FF 23,900–142,100 depending on the risk of hospitalisation in the patient population studied. Another study in a French setting found fluvoxamine to be cost-effective as maintenance treatment in recurrent depression (39). Nuijten (40) assessed the cost-effectiveness of implementing the Dutch clinical treatment guidelines of continuation treatment in major depression, and concluded that continuation treatment ought to be extended to maintenance treatment to reach levels of cost per QALY gained considered to be cost-effective. Kamlet et al. (8) came to the conclusion that antidepressant maintenance therapy for 3 years is cost-saving when compared with placebo or interpersonal therapy alone in the area of Pittsburgh in the USA. Despite a rather small number of previous assessments of the cost-effectiveness of maintenance treatment in recurrent depression internationally, our study adds to a growing understanding that maintenance treatment is not only effective in preventing episodes in recurrent depressive disorder, but also beneficial from an economical point of view.

Our sensitivity analysis shows that the most critical parameter for the ICER is the maintenance treatment period. We reach an ICER above $80,000 with a short prophylactic treatment period (6 months), whereas it decreases with longer treatment period and follow-up. In a sensitivity analysis, productivity costs because of reduced performance while at work were included based on American findings (24). This analysis resulted in cost-savings in favour of prophylactic treatment. The base-case analysis in the present work, did not allow for repeated relapses/recurrences within the time frame of the analysis. Allowing for these consequences did, however, only increase the ICER marginally. The probabilistic sensitivity analysis performed showed that the cost-effectiveness results for venlafaxine were stable for reasonable variations in key parameters.

There are, however, a number of limitations with the current analysis, which should be considered when interpreting the results presented. First, the current analysis is based on effect data from a multicentre clinical trial, and there are several concerns when applying clinical effects of a treatment from one setting to another. Second, the health economic data employed in the current analysis was based on an observational study carried out in Swedish primary care settings. Although the characteristics of the patients in the clinical trial matched well with the patients included in the Swedish observational study, we might underestimate costs of hospitalisation, as relatively few patients were hospitalised in that study. As a consequence of this underestimation, our cost-effectiveness results are probably conservative. Third, the analysis of the cost-effectiveness was based on a fairly short follow-up period, and more longitudinal follow-up studies are needed to be able to evaluate the cost-effectiveness of maintenance treatment over a longer time period than 2 years. Fourth, venlafaxine was evaluated compared with placebo in this study, which was appropriate as the optimal duration of maintenance treatment is still an issue for debate. Whether venlafaxine would be cost-effective compared also to other antidepressants in the maintenance phase was beyond the scope of this study, as it would require direct comparative data during the maintenance phase, or at least long-term data from a comprehensive meta-analysis. Investigation of this issue is left for further research.

## Conclusions

A growing body of literature and international treatment guidelines argue for preventive treatment in recurrent depressed patients. Based on a recent clinical trial, our results indicate that maintenance treatment for 2 years in recurrent depression is cost-effective in the Swedish treatment setting.
